# The Combination of Homocysteine and C-Reactive Protein Predicts the Outcomes of Chinese Patients with Parkinson's Disease and Vascular Parkinsonism

**DOI:** 10.1371/journal.pone.0019333

**Published:** 2011-04-27

**Authors:** Limin Zhang, Junqiang Yan, Yunqi Xu, Ling Long, Cansheng Zhu, Xiaohong Chen, Ying Jiang, Lijuan Yang, Lianfang Bian, Qing Wang

**Affiliations:** Department of Neurology, The Third Affiliated Hospital of Sun Yat-Sen University, Guangzhou, Guangdong, People's Republic of China; Georgia Health Sciences University, United States of America

## Abstract

**Background:**

The elevation of plasma homocysteine (Hcy) and C-reactive protein (CRP) has been correlated to an increased risk of Parkinson's disease (PD) or vascular diseases. The association and clinical relevance of a combined assessment of Hcy and CRP levels in patients with PD and vascular parkinsonism (VP) are unknown.

**Methodology/Principal Findings:**

We performed a cross-sectional study of 88 Chinese patients with PD and VP using a clinical interview and the measurement of plasma Hcy and CRP to determine if Hcy and CRP levels in patients may predict the outcomes of the motor status, non-motor symptoms (NMS), disease severity, and cognitive declines. Each patient's NMS, cognitive deficit, disease severity, and motor status were assessed by the Nonmotor Symptoms Scale (NMSS), Mini-Mental State Examination (MMSE), the modified Hoehn and Yahr staging scale (H&Y), and the unified Parkinson's disease rating scale part III (UPDRS III), respectively. We found that 100% of patients with PD and VP presented with NMS. The UPDRS III significantly correlated with CRP (*P* = 0.011) and NMSS (*P* = 0.042) in PD patients. The H&Y was also correlated with Hcy (*P* = 0.002), CRP (*P* = 0.000), and NMSS (*P* = 0.023) in PD patients. In VP patients, the UPDRS III and H&Y were not significantly associated with NMSS, Hcy, CRP, or MMSE. Strong correlations were observed between Hcy and NMSS as well as between CRP and NMSS in PD and VP.

**Conclusions/Significance:**

Our findings support the hypothesis that Hcy and CRP play important roles in the pathogenesis of PD. The combination of Hcy and CRP may be used to assess the progression of PD and VP. Whether or not anti-inflammatory medication could be used in the management of PD and VP will produce an interesting topic for further research.

## Introduction

Idiopathic Parkinson's disease (PD) is the second most common neurodegenerative disorder following Alzheimer's disease; however, attention has recently been drawn to vascular parkinsonism (VP). Inflammatory mechanisms have been recognized to play important roles in the pathogenesis and development of PD and VP, and various inflammatory biomarkers have been investigated as potential predictors of PD and VP. Several lines of research have shown that serum oxidative stress and pro-inflammatory cytokines such as interleukin (IL)-1b, IL-2, IL-6, HLA-DRB1, phospholipase A(2), tumor necrosis factor (TNF)-a, and TNF-a receptors are elevated in the brain and cerebral spinal fluid of PD patients [1], [2], [3], [4].

In addition to those inflammatory related mediators, plasma C-reactive protein (CRP) and homocysteine (Hcy) levels have recently attracted wide clinical attention as predictive of PD progression. One clinical study by Song indicated that CRP levels in an early PD group were higher than those of healthy controls [5]. A similar result has been observed in another study, which demonstrated an elevated serum CRP in PD and ischemic cerebrovascular disease compared to control subjects [6]. It has been documented that the plasma concentration of Hcy is elevated in PD patients, and this increase is associated with the long-term effects of chronic or acute administration of L-dopa [7], [8], [9], [10]. Investigations on the association of Hcy with PD dementia (PDD) have shown conflicting results [11]; some studies did not observe a direct relationship between Hcy plasma levels and cognitive impairment and dementia in PD [7], [12], while others found a significant correlation of hyperhomocysteinemia with the presence of dementia in PD [8], [13]. However, to the best of our knowledge, few studies have been performed to investigate the combined effects of Hcy and CRP in PD and VP.

PD patients are characterized by typical motor symptoms (MS), such as bradykinesia, resting tremor, and rigidity, as well as non-motor symptoms (NMS) [14], which are also highly prevalent. Patients normally show an increasing severity of NMS as the disease develops, although some of the NMS, such as depression and impaired cognition, may occur in the premotor stage of this disease [15]. Whether the severity and progression of PD and VP, as measured by MS and NMS, could be evaluated by the combination of Hcy and CRP is an interesting topic. We undertook this study to determine whether plasma Hcy and CRP levels in patients with PD and VP are associated with the development of the diseases and their cognitive dysfunctions. The primary aim of this study was to use different clinical parameters to compare PD and VP and to describe the correlation between the symptom scores and plasma Hcy and CRP levels in patients with PD and VP. An additional aim was to assess whether Hcy and CRP in PD and VP are associated with poor motor function and L-dopa dosage. A third aim of this study was to conduct an exploratory analysis to identify the association of NMSS domains and plasma Hcy/CRP levels.

## Methods

### Patients

This cross-sectional study was carried out at the Department of Neurology of the Third Affiliated Hospital of Sun Yat-sen University, Guangzhou, P. R. China. From July 2009 to December 2010, 43 patients (25 males and 18 females) with PD and a mean age (± SD) of 58.9±13.7 (Table 1) were recruited from the inpatient/outpatient clinic. The patients were identified according to the United Kingdom PD Society Brain Bank (UK-PDSBB) criteria for the diagnosis of idiopathic PD [16]. In addition, 45 patients (23 males and 22 females) with VP and a mean age (± SD) of 67.0±11.6 (Table 1) were recruited according to the Zijlmans' criteria for the diagnosis of VP [17].

**Table 1:** Demographic, motor, and non-motor parameters.

Clinical parameters		PD			VP		
		Mean(SD)	Min	Max	Mean(SD)	Min	Max
**Gender (n)**	Male n(%)	25(58.1)			23(51.1)		
	Female n(%)	18(41.9)			22(49.9)		
**Age (years)**		*58.9(13.7*4)	40	83	67.0 (11.6)	45	83
**Disease duration (years)**		6.2(3.7)	0.08	26	5.8(4.2)	0.06	14
**H&Y**		2.39(1.09)	1	6	2.34(1.28)	1	4
**Daily L-dopa dosage (mg)**		392.3(225.3)	200	800	383.3(189.9)	200	800
**BMI**		22.80(2.12)	19.22	25.39	21.68(2.37)	18.32	25.39
MMSE		25.32(2.18)	21	30	22.08(2.93)	18	30
UPDRS(III)		17.89(5.65)	6	29	19.23(10.60)	8	33
NMSS(total)		131.14(9.24)	47	219	135.57(48.76)	41	235
	Cardiovascular	2.21(3.87)	0	9	2.64(2.31)	0	8
	Sleep/Fatigue	28.14(3.92)	0	46	26.57(10.11)	0	46
	Mood	27.14(13.31)	0	58	26.43(12.38)	0	55
	Perceptual problem	7.36(3.42)	0	22	9.71(7.67)	0	26
	Attention/memory	13.29(6.60)	0	22	13.07(7.42)	0	21
	Gastrointestina	11.57(9.15)	0	32	12.57(8.78)	0	31
	Urinary	13.21(9.93)	0	32	12.36(8.10)	0	36
	Sexual function	13.64(5.0)	0	24	15.86(6.8)	0	24
	Miscellaneous	15.64(10.72)	0	36	16.36(9.79)	0	38

Abbreviations: SD, Standard deviation; BMI, Body Mass Index; MMSE, mini-mental state examination; UPDRS III, the unified Parkinson's disease rating scale part III; H&Y, the modified Hoehn and Yahr staging scale; and NMSS, non-motor symptoms scale for Parkinson's disease (range of possible scores from 0 to 360).

### Ethics Statement

The local Ethics Committee of Third Affiliated Hospital of Sun Yat-sen University approved the study, which has been conducted according to the principles expressed in the Declaration of Helsinkiand. The patients gave informed consent for the investigation. This consent was verbal for those measurements on MMSE,H&Y,UPDRS(III), and NMSS in this study. All those measurements were anonymously in this study. Since those measurements benefit to the therapies of PD and VP patients involved in this study, the ethics committee specifically approved that procedure and wish those measurements become a routine way for the treatment of PD and VP patients in the future. The consent for the measurement of plasma homocysteine and C-reactive protein was written.

### Study Design

Experienced neurologists performed the evaluations and completed neurological examinations of the inpatients and outpatients. All patients with PD in this study fulfilled the criteria of the UK-PDSBB [16]. The exclusion criteria were as follows: (1) PD patients with disability due to neurological disorders other than PD, such as cerebrovascular disease, sequelae, or psychosis [18]; (2) PD patients with somatic diseases that could have a potential effect on NMS (e.g., pain syndromes, advanced diabetes mellitus, malignancy, renal, hepatic or heart failure, severe anemia, or any other acute or chronic debilitating or life-threatening disease/state) [18]; (3) PD patients with moderate/severe cognitive impairment, as determined by a Mini-Mental State Examination (MMSE) (the Chinese version of the MMSE) score lower than the median value determined in a reference population-based sample of corresponding age and education [15], [19]; and (4) individuals who refused to participate in the study. All patients with VP in this study fulfilled the criteria according to Benamer and Zijlmans' reports [17], [20] as follows: (1) parkinsonism, defined as bradykinesia, and at least one of the following: rest tremor, rigidity or postural instability; (2) cerebrovascular disease, defined as evidence of relevant cerebrovascular disease by brain imaging or the presence of focal signs or symptoms that are consistent with stroke; and (3) a relationship between (1) and (2): an acute or delayed progressive onset of parkinsonism (within 1 year) after stroke with evidence of infarcts in or near areas that increase the basal ganglion motor output or decrease the thalamocortical drive directly, *or* an insidious onset of parkinsonism with extensive sub-cortical white matter lesions, bilateral symptoms at the onset and the presence of early shuffling gait or early cognitive dysfunction. All subjects completed the following battery of standard assessment measures: a standard demography form, the unified Parkinson's disease rating scale part III (UPDRS III- motor) [21] and the modified Hoehn and Yahr staging scale (H&Y) [22]. The UPDRS III-motor and H&Y scales were used to evaluate motor dysfunctions and disease severity. The degree of NMS in every patient was measured by the NMSS [23]. Cognitive abilities were evaluated with the MMSE [19]. All scales were available and validated for the Chinese population. The demographics and clinical data of the subjects are shown in Table 1.

Venous blood samples for CRP and Hcy measurement were obtained from all subjects in the study. The serum was separated within the hour by centrifugation at 3,000 rpm for 10 min, and the separated sera were stored at −70°C until the laboratory evaluation.The CRP level was measured by latex immunoturbidimetric assay according to Ichihara's study [24], and the plasma level of Hcy was determined using a solid phase competitive chemiluminescent enzyme immunoassay [7].

### Statistical analysis

All data for the continuous variables (age, disease duration, daily levodopa dosage, UPDRS III, MMSE, NMSS, Hcy, and CRP) are shown as the means ± standard deviation, and the categorical variable (gender) is shown as a percentage.The total scores of UPDRS III, MMSE, NMSS, Hcy, and CRP were calculated by summing single items. The strength of the association for correlation coefficients was interpreted as follows: ≤0.19, negligible; 0.20 to 0.39, weak; 0.40 to 0.59, moderate; 0.60 to 0.79, strong; and ≥0.80, very strong [25]. Student's t-test was used to compare the differences of age, Hcy, CRP, BMI, MMSE, UPDRS III, and NMSS between PD and VP patients. A Spearman's rank correlation coefficient was used to evaluate the association among UPDRS III and Hcy/CRP, UPDRS III and MMSE/NMSS, nine different NMSS domains and Hcy/CRP, Hcy/CRP and H&Y, H&Y and MMSE/NMSS, Hcy/CRP and L-dopa dosage, and Hcy/CRP and disease duration. The variables tested included age, disease duration, H&Y, MMSE, NMSS, UPDRS III, Hcy and CRP. SPSS 13.0 software (Chicago, IL, USA) was used for the statistical analyses. *P* values of less than 0.05 were regarded as statistically significant.

## Results

### Patient characteristics

In total, 88 PD subjects, including 43 PD patients [25 males (58.1%) and 18 females (41.9%)] and 45 VP patients [23 males (51.1%) and 22 females (49.9%)], were enrolled in this cross-sectional study. The mean ages of the PD and VP patients were 58±13.74 years (range, 43 to 83) and 67±11.6 years (range, 45 to 83), respectively. The mean durations of PD and VP symptoms were 6.2±3.7 years (range, 0.08 to 26) and 5.8±4.2 years (range, 0.06 to 14), respectively. The H&Y stages ranged from 1 to 4 for patients with PD and VP. With respect to the levodopa (L-dopa) therapy, the average dosages were 392.3 mg and 383.3 mg daily for PD and VP, respectively. Details of the demographics of all 88 patients are listed in Table 1.

The mean age for patients with VP was higher than that in the PD patients (67.03 vs. 58.88, *P* = 0.006), and the MMSE for patients with VP was lower than that in PD patients (22.08 vs. 25.32, *P* = 0.007, Table 2). No significant difference in the Hcy, CRP, BMI, UPDRS III, or nine dimensions of NMSS was found between the PD and VP patients.

**Table 2:** Comparison of age, BMI, Hcy, CRP, UPDRS (III), MMSE, and NMSS between PD and VP.

Variable		PD	VP	*T* value	*P* value
**Age**		58.88±13.7	67.03±11.58	2.809	0.006*
**Hcy**		14.08±0.77	15.12±0.90	0.087	0.39
**CRP**		2.06±0.28	2.59±0.39	1.136	0.26
**BMI**		22.80±2.12	21.68±2.37	1.247	0.224
**MMSE**		25.32±2.18	22.08±2.93	2.955	0.007*
**UPDRS(**III**)**		17.89±5.65	19.23±10.60	0.389	0.701
**NMSS(total)**		131.14±9.24	135.57±48.76	0.671	0.508
	Cardiovascular	2.21±2.39	2.64±2.31	−0.483	0.633
	Sleep/Fatigue	28.14±9.69	26.57±10.11	0.420	0.678
	Mood	27.14±13.31	26.43±12.38	0.144	0.886
	Perceptual problem	7.36±7.06	9.71±7.67	−0.846	0.405
	Attention/memory	13.29±6.60	13.07±7.56	0.081	0.936
	Gastrointestina	11.57±9.75	12.57±8.78	−0.285	0.778
	Urinary	13.21±7.91	12.36±8.10	0.283	0.779
	Sexual function	13.64±6.29	15.88±6.8	−0.894	0.380
	Miscellaneous	15.64±10.72	16.36±9.79	−0.192	0.849

* *P*<0.01.

Abbreviations: SD, Standard deviation; BMI, Body Mass Index; MMSE, mini-mental state examination; UPDRS III, the unified Parkinson's disease rating scale part III; and NMSS, non-motor symptoms scale for Parkinson's disease (range of possible scores from 0 to 360).

When the PD and VP subjects were divided into two groups (age≤60,age>60), the levels of CRP in the PD patients above 60 years old were significantly higher than those in the PD patients younger than 60 years old (3.04 vs.1.47, P = 0.005, Table 3), and the levels of Hcy in VP patients above 67 years old were significantly higher than those in VP patients younger than 67 years old (16.79 vs. 12.78, P = 0.009, Table 3)

**Table 3:** Comparison of Hcy and CRP among patients with PD and VP.

Variable	PD			VP		
	Age≤60	Age>60	*P*	Age≤67	Age>67	*P*
Hcy	14.50±5.82	13.65±3.18	0.604	12.78±3.88	16.79±5.55	0.009*
CRP	1.47±0.96	3.04±2.21	0.005*	3.31±0.91	3.27±0.82	0.97

* *P*<0.01

### Correlations between UPDRS III and Hcy/CRP, UPDRS III and MMSE, NMSS domains and Hcy/CRP, Hcy/CRP and H&Y, Hcy/CRP and L-dopa dosage, Hcy/CRP and disease duration, and Hcy/CRP and MMSE

There was a significant correlation between UPDRS III and CRP (r_S_ = 0.656, *P* = 0.011, Table 4) as well as between UPDRS III and NMSS (r_S_ = 0.550, *P* = 0.042, Table 4) in patients with PD. Notably, the H&Y was also correlated with Hcy (r_S_ = 0.759, *P* = 0.002, Table 4), CRP (r_S_ = 0.852, *P* = 0.000, Table 4), and NMSS (r_S_ = 0.602, *P* = 0.023, Table 4) in patients with PD. In the VP patients, the UPDRS III and H&Y were not significantly associated with NMSS, Hcy, CRP, or MMSE. Significant correlations were observed between Hcy and NMSS in PD and VP patients (r_S_ = 0.795/842, *P* = 0.001/0.000/<0.001, respectively, Table 5). Similarly, the CRP and NMSS also strongly correlated with NMSS (r_S_ = 0.781/699, *P* = 0.001/0.005/<0.001, respectively, Table 5). Specifically, when evaluating the correlation between Hcy and the different NMSS dimensions in PD and VP, significant correlations, from low to high, were observed in miscellaneous (*P* = 0.042 for VP), urinary (*P* = 0.009 for PD), mood (*P* = 0.009 for VP), urinary (*P* = 0.005 for VP), mood (*P* = 0.001 for PD), and sleep/fatigue (*P*<0.000 for VP). Similarly, when evaluating the correlation between CRP and the different NMSS dimensions in PD and VP, significant correlations, from low to high, were observed in mood (*P* = 0.029 for VP), sleep/fatigue (*P* = 0.013 for VP), urinary (*P* = 0.01 for PD), miscellaneous (*P* = 0.007 for VP), gastrointestinal (*P* = 0.004 for PD), urinary (*P*<0.002 for VP), and mood (*P* = 0.001 for PD). For the remaining four dimensions, no significant correlations were found (Table 5). In addition, Hcy/CRP were significantly associated with L-dopa dosage for both PD and VP patients (*P*<0.001, Table 6), and Hcy/CRP were correlated with H&Y (*P*<0.01, Table 6) in PD patients. No significant correlations were found between Hcy/CRP and MMSE and Hcy/CRP and disease duration in patients with PD.

**Table 4:** Spearman's rank correlation coefficient (r_s_) and *P* values between UPDRS III and clinical variables, and between H&Y and clinical variables.

Variable	UPDRS(III) (PD)		H&Y (PD)		UPDRS(III) (VP)		H&Y (VP)	
	r	*P*	r	*P*	r	*P*	r	*P*
**Hcy**	0.463	0.096	0.759	0.002	0.216	0.459	0.637	0.014*
**CRP**	0.656	0.011*	0.852	0.000**	0.013	0.964	0.453	0.104
**BMI**	−0.053	0.858	0.191	0.514	0.132	0.654	0.431	0.124
**MMSE**	−0.308	0.284	−0.322	0.262	−0.203	0.486	−0.522	0.056
**NMSS (total)**	0.550	0.042*	0.602	0.023*	0.318	0.268	0.735	0.040*

* *P*<0.05,

** *P*<0.001.

Abbreviations: r_s_, Spearman's rank correlation coefficient; H&Y, the modified Hoehn and Yahr staging scale; UPDRS III, the unified Parkinson's disease rating scale part III; MMSE, mini-mental state examination; NMSS, non-motor symptoms scale for Parkinson's disease.

**Table 5:** Spearman's rank correlation coefficient (r_s_) and *P* values between NMSS domains and Hcy, and between NMSS domains and CRP.

Variable	Hcy (PD)		CRP (PD)		Hcy (VP)		CRP (VP)	
	*r*	*p*	*r*	*p*	*r*	*P*	*r*	*P*
**NMSS (total)**	0.795	0.001**	0.781	0.001**	0.842	0.000***	0.699	0.005**
**Cardiovascular**	0.503	0.067	0.474	0.087	0.284	0.326	0.178	0.543
**Sleep/Fatigue**	0.478	0.084	0.477	0.084	0.854	0.000***	0.642	0.013*
**Mood**	0.767	0.001**	0.801	0.001**	0.666	0.009**	0.581	0.029*
**Perceptual problem**	0.528	0.052	0.649	0.012*	0.496	0.071	0.257	0.375
**Attention/memory**	0.232	0.424	0.224	0.440	0.331	0.248	0.134	0.647
**Gastrointestina**	0.485	0.078	0.722	0.004**	0.540	0.046*	0.349	0.221
**Urinar**	0.668	0.009**	0.660	0.010*	0.702	0.005**	0.740	0.002**
**Sexual function**	0.299	0.299	0.162	0.580	0.471	0.089	0.396	0.161
**Miscellaneous**	0.461	0.067	0.133	0.650	0.548	0.042*	0.683	0.007**

* *P*<0.05,

** *P*<0.01,

*** *P*<0.001.

Abbreviations: r_s_, Spearman's rank correlation coefficient; NMSS, non-motor symptoms scale.

**Table 6:** Spearman's rank correlation coefficient (r_s_) and *P* values between Hcy and clinical variables, and between CRP and clinical variables.

Variable	Hcy (PD)		CRP (PD)		Hcy (VP)		CRP (VP)	
	*r*	*p*	*r*	*p*	*r*	*P*	*r*	*P*
**Hcy**	1		0.912	0.000***	1		0.686	0.007**
**CRP**	0.912	0.000***	1		0.686	0.007**	1	
**Daily L-dopa dosage**	0.814	0.000***	0.693	0.006**	0.854	0.000***	0.698	0.005**
**H&Y**	0.759	0.002**	0.852	0.000***	0.637	0.014*	0.453	0.104
**Disease duration**	0.460	0.098	0.447	0.109	0.531	0.051	0.616	0.019*
**MMSE**	−0.431	0.124	−0.387	0.171	−0.298	0.301	−0.313	0.276

* *P*<0.05,

** *P*<0.01,

*** *P*<0.001.

Abbreviations: r_s_, Spearman's rank correlation coefficient; H&Y, the modified Hoehn and Yahr staging scale; MMSE, mini-mental state examination.

## Discussion

PD is the second most common neurodegenerative disorder following AD and is characterized by a disturbance of the central dopaminergic system; the concept of VP was first mentioned by Critchley in 1929 [29]. Over the past few years, correlations between VP and PD patients have received significant attention in the diagnosis, therapy, and evaluation of these two diseases [17], [26], [27], [28]. The primary goals of this study were to compare Chinese patients with PD and VP by systematically collecting information about demographics, such as age and disease duration, motor and non-motor dysfunctions, and the serum levels of CRP and Hcy. To the best of our knowledge, this report is the first to evaluate the association between motor/non-motor effects and CRP/Hcy in Chinese patients with PD and VP using unified and integrated scales (NMSS, H&Y, and MMSE).

In this study, the VP group presented many characteristics, such as resting tremor, postural instability, and rigidity, that are similar to those described in the original VP report by Critchley [29]. We found that NMS (including cardiovascular, sleep, and mood disorders) were very common in Chinese patients with PD and VP, with a prevalence of NMS in the group being 100%, and the NMSS for PD (mean = 131.14, Table 1) was consistent with a previous report [15]. However, we did not find a significant difference in NMS between PD and VP, which suggests that NMS equally influenced PD and VP in Chinese subjects. Interestingly, as shown in Tables 1 and 2, our patients with VP were significantly older than those patients with PD, and a shorter duration of symptoms in the VP group was also observed. This finding has also been documented in some other VP studies [26], [27], and it implies that Chinese VP subjects developed symptoms later in life but experienced deterioration faster than the PD patients. Additionally, a later age at onset of VP would favor a vascular cause. Interestingly, a significant reduction of the MMSE was observed in VP patients when compared to the PD group (mean = 25.32 for PD, mean = 22.08 for VP, P = 0.007, Table 2). This result demonstrated that compared to PD subjects, Chinese VP patients had already undergone a greater decline in cognitive function by the time they visited a neurologist. This result is in agreement with Zijlmans' point [17] and implies that our VP patients may have more subcortical lesions than patients with PD and that this subcortical vascular cause may have led to the declined cognition.

Recent studies have shown that inflammatory responses are observed in the nigrostriatal regions of dopaminergic degeneration, and some protective strategies against inflammatory development occur when anti-inflammatory medications are taken. Among those inflammatory mediators, Hcy and CRP have received substantial attention for the past 15 years as having an important role in and potentially as risk factors for PD. An elevated plasma Hcy level represents a risk factor for declined cognition and dementia in the general population [30], and it has been well documented to be associated with mild cognitive impairment (MCI), Alzheimer's disease (AD), PD, and vascular dementia [7], [11], [31]. Similarly, increased plasma CRP is correlated to myocardial infarction, PD, and ischemic stroke [6], [32]. However, limited studies have investigated the association of the combination of Hcy and CRP in PD and VP subjects. To clarify, to determine if Hcy and CRP contribute to the motor dysfunction and cognitive decline in VP and PD patients, we examined the plasma Hcy/CRP and compared their levels in PD and VP patients. Our data did not show a significant difference in Hcy and CRP between the PD and VP groups (Table 2). This result demonstrates that the inflammatory mediators Hcy and CRP might contribute equally to the progression of these two diseases. Interestingly, when we separated the subjects into two groups according to age (<60 and >60), we noticed that the CRP levels in PD subjects above 60 years old were much higher than those patients under 60, whereas the Hcy levels in VP subjects above 60 were less than those under 60 (Table 3). Two reasons probably led to the discrepancy in the CRP and Hcy levels in patients with PD. First, as a neurodegenerative disease, PD exacerbates with age, and the inflammatory process aggravates the progression in PD patients above 60 when compared to patients under 60. This exacerbated inflammatory response in PD patients above 60 can be reflected in the obviously elevated levels of CRP, a biomarker of systemic inflammation. Second, Hcy, though recognized as an indicator of neurotoxic mediator, is profoundly affected by several factors, such as some B-vitamin (folate, and B12, and B6) deficiencies, genetic polymorphisms of MTHFR and CßS, and L-dopa intake [11], [33]. These influences may have lead to the unchanged level of Hcy between PD patients above and under 60 in this study. We did not observe a significantly changed level of CRP, but we did observe a significant upregulation of Hcy in VP patients above 67 when compared with those under 67. The precise reasons remain unclear, but it may reflect a different pathogenesis for PD and VP, the latter of which mainly results from subcortical vascular causes; additionally, Hcy is obviously increased in cerebral-vascular diseases [28], [33], [34], [35]. Our findings suggest that the chronic inflammatory response may be worse in older PD patients and that an anti-inflammatory strategy would be useful in the management of PD.

Spearman's rank correlation coefficients were determined to explore the association of UPDRS III with the Hcy, CRP, H&Y, MMSE and NMSS in PD and VP. Similar to our previous report [15], highly significant correlations were observed between motor dysfunctions and NMS burden in patients with PD (r_S_ =  0.550, P = 0.042, Table 4). This result suggests that both disease severity and motor dysfunctions may influence NMS in PD and that NMS may have multiple effects on the motor complications in PD. Interestingly, no correlations were found between the UPDRS III and Hcy in PD (r_S_ =  0.463, P = 0.096, Table 4) and VP (r_S_ =  0.216, P = 0.459, Table 4) or between the UPDRS III and CRP in VP (r_S_ =  0.013, P = 0.964, Table 4). However, similar to the result in Table 3, the inflammatory mediator CRP was closely correlated to UPDRS III in the PD group (r_S_ = 0.656, P = 0.011, Table 4), further demonstrating that the inflammatory response in PD patients may contribute to motor dysfunction [36].

To further correlate the association of the individual domains of NMS with Hcy, CRP, and H&Y, we identified that some dimensions of NMS in PD and VP significantly correlated with the Hcy and CRP (Table 5) using the Spearman's rank correlation coefficient analysis. Clinical associations of elevated CRP levels in PD and VP patients have not, to our knowledge, previously been described. Although our results (Table 5) indicate the percent variation, they only explain the r_S_ separately and did not take into account the contributions of the other domains. Significant correlations (Table 4) were also observed between the H&Y and NMSS in both PD and VP. This result is consistent with our previous study [15] and implies that the disease severity for both PD and VP influences non-motor dysfunctions. In addition, these data show that Hcy and CRP are highly correlated with NMS in both PD and VP patients (Table 5). Specifically, Hcy and CRP have robust association with mood and urinary domains, further demonstrating that these two dysfunctions would be important targets for evaluation by plasma levels of Hcy and CRP. Consistent with our notions, one study by O'Suilleabhain also reported that a high level of Hcy was correlated to mood deterioration [37].

In our study, the associations between Hcy/CRP and L-dopa, H&Y, the disease duration, and the MMSE in PD and VP were investigated. Profound associations were observed between Hcy and L-dopa dosage as well as between Hcy and H&Y in PD and VP patients (Table 6). This result is consistent with previous reports [11], [37], [38], [39], and it may imply that levodopa drains methylation reserves: methylation of levodopa and dopamine converts the methyl donor methionine into Hcy [37]. The L-dopa related plasma Hcy may contribute to the progression of PD/VP and the development of motor dysfunction, which is reflected in the observed significant correlation between the H&Y score and Hcy (Table 6). Similarly, significant associations between CRP and L-dopa dosage as well as between CRP and H&Y in PD were obtained. This result suggests that as an inflammatory biomarker, CRP might be used to predict the severity or outcome in PD patients. Interestingly, no significant correlation was obtained between Hcy/CRP and the MMSE in either PD or VP, implying that Hcy and CRP plasma levels did not predict the cognitive status of PD and VP patients. This result is consistent with Barone and Rodriguez-Oroz's studies, which showed a similar degree of cognitive impairment in PD patients with low and high Hcy levels [7], [40].

Several limitations of this study should be noted: (1) only a small number of PD patients (88 patients) were recruited, and the disease durations were relatively short (3.7 years for PD and 4.2 years for VP); (2) the UPDRS III scale had a low mean score (mean  = 17.89 for PD, 19.23 for VP); (3) mainly PD patients in the early stages of disease were enrolled, as indicated by the low median stage of the H&Y scale and the relatively high MMSE score (25.32 for PD, 22.08 for VP); (4) genetic factors such as the *MTHFR* genotype and folate administration were not considered in this study; and (5) to validate and complete the questionnaire, we only chose PD and VP subjects with sufficient cognitive ability, significantly narrowing the population in this study. The population chosen according to the above (1)-(4) in this study may produce a bias for Hcy and CRP levels in PD and VP patients. Therefore, larger studies need to be done, expanding this work to a broader population. In addition, different stages of PD patients in both UPDRS III and H&Y scores should be included to compensate for current shortcomings.

In conclusion, our findings support the hypothesis that as exquisitely sensitive systemic mediators of inflammation, Hcy and CRP play important roles in the pathogenesis of PD. To our best knowledge, this report is the first to combine the plasma levels of Hcy and CRP to assess the progression of PD and VP. The combination of Hcy and CRP, especially CRP, may predict the outcomes of non-motor dysfunctions in PD and VP. A causal link in either direction is feasible; a systemic Hcy and CRP change may predispose a person to PD or VP or otherwise impair dopaminergic neuronal function. Alternatively, worse parkinsonism may predispose a person to the alteration of Hcy and CRP due to some medication factors, such as L-dopa treatment. Our findings, if confirmed, indicate that disease burden in patients with PD and VP, such as poor motor performance, disease severity, and urinary dysfunction, is associated with plasma Hcy and CRP levels. Whether or not anti-inflammatory medication could be used in the management of PD and VP will produce interesting work in the future.
